# Editorial: The representation of psychiatry and mental health in popular culture

**DOI:** 10.3389/fpsyt.2024.1432346

**Published:** 2024-05-20

**Authors:** Hua Wang, Ian Cummins, Yukari Seko

**Affiliations:** ^1^ Department of Communication, University at Buffalo, The State University of New York, Buffalo, NY, United States; ^2^ School of Health & Society, University of Salford, Salford, United Kingdom; ^3^ School of Professional Communication, Toronto Metropolitan University, Toronto, ON, Canada

**Keywords:** media representation, psychiatry, mental illness, popular culture, stigma, discrimination

Mental illness has a complicated history in popular culture (1). Stories about the “madman,” whether fictional or real, often unfold in dark, unpredictable, and violent narratives, contributing to moral panics and cultural paranoia in society (1–4). These representations profoundly shape the public perceptions, attitudes, and behaviours toward psychiatry and mental health (1, 5–7). This Research Topic aims to investigate media representations of mental health through diverse conceptual perspectives and analytical approaches.

The six articles in this Research Topic covered popular entertainment and prominent cases across the UK, USA, and Belgium, spanning various media forms like newspapers, magazines, television series, and video games. Employing social scientific methods and critical cultural studies, the authors examined phenomena regarding the creative industry, mainstream media, and policies directly and indirectly affecting perceptions and treatment of mental illness. Topics included discrimination and stigma associated with disorders such as schizophrenia, personality disorders, PTSD, depression, and anxiety, alongside intersections with racial trauma and socioeconomic disparities, as well as adverse consequences like imprisonment and suicide.

De Hart et al. examined the news coverage of Belgium’s first criminal case since euthanasia for psychiatric patients became legal. Using both quantitative and qualitative content analysis, they found predominantly neutral news coverage regardless of ideological backgrounds and publication platforms, though subtle differences emerged in content selection and tonality. The end-of-life decision-making of a 38-year-old woman with borderline personality disorder and/or autism ignited wider legal and ethical debates on euthanasia for incurable mental illness and unbearable suffering, including the problematic nature of defining psychiatric suffering.

Cummins critiqued the British news media’s recurrent use of a photograph of Christopher Clunis, a young Black man with a history of mental illness, following his conviction of manslaughter of a White stranger at a London train station in 1992. Clunis’ death in February 2021, announced to the public 22 months later, prompted Cummins to argue how the Clunis image symbolised negative racial stereotypes associated with madness and mentally ill, contributing to moral panics and representing the failings of psychiatric policy and community care in the late 20th century.

Wang et al. investigated news coverage of the controversial Netflix original series, *13 Reasons Why*, upon its Season 1 premiere in 2017. Their quantitative content analysis of 3,150 sentences across 97 articles revealed that content directly addressing mental health themes were more negative than positive, especially concerns for glamorising teen suicide, the copycat effect, and portraying adults as inept and uncaring. Despite some praise for raising awareness of the reality of young people struggling with mental illness, most failed to comply with the WHO guidelines.

Tenzek et al. studied mental health portrayals in an award-winning American drama series, *This Is Us*, focusing on anxiety storylines of a main character Randall. Their narrative analysis of 38 episodes underscored the importance of chronic care and various forms of literacy (i.e., English, personal health, and mental health) for effective depictions of mental illness. They recommended mental health professionals to use Randall’s narrative as an entertainment-education tool to discuss chronic care delivery and promote literacy.

Quadros et al. analysed the 2024 British Academy Television Awards (BAFTA) best drama series, Top Boy. Its main character, Dushane “Top Boy” Hill, runs a gang operation for narcotics distribution out of a fictional housing estate in the London borough of Hackney. The authors commended the series for depicting a broad range of mental health themes, exploring the intersectionality of syndemics involving racial trauma, social inequities, PTSD, substance misuse, and gang violence. They highlighted the show’s broad audience reach and educational potential.

Buday et al. examined 456 best-selling UK-based video games from 2002 to 2021, revealing mental illness representation in 54 (12%) games, with schizophrenia being the most frequent condition. Of 57 such portrayals, 43 (75%) were negative, 13 (23%) were neutral, and only one was positive. Furthermore, only 13 (3%) games included any form of psychiatric or psychological intervention, and sadly no positive portrayals were found. The authors emphasized the overlooked adverse impacts of video games compared to the film industry.

The conceptual arguments and research findings in this Research Topic are largely consistent with prior literature and complementary to recent work in other domains (e.g., 8–10). Taken together, they remind us that individuals with mental illness are embedded in communities and systems, and it is the dynamics across micro-, meso-, and macro-level factors that shape the suffering and possibilities of positive change (11).

The editorial process of this Research Topic also revealed research gaps and future opportunities. First, although the authors shared critical cases on women, youth, and Black communities living with mental illness in Western societies, there is a scarcity of rigorous studies at the intersection of popular culture, media representation of psychiatry and mental health, and vulnerable and marginalised groups worldwide, particularly non-Western perspectives.

Second, while one article explored mental illness representation in video games, our constantly evolving popular culture provides a fertile ground for research. Future studies can explore, for example, music, anime, digital games, AR/VR/XR, social media, AI applications, and the role of celebrities for public engagement and health communication (1, 9, 10, 12–15).

Third, while we appreciate the authors’ use of content analysis, narrative analysis, and critical analysis in this Research Topic, additional methodologies such as systematic reviews, meta-analyses, experiments, and computational research can further enrich our knowledge.

Finally, opportunities abound for empirical examinations to facilitate positive change in policies and practices across the entertainment industry, journalism, social services, and legal systems. The stories we tell can shape who we are. By focusing on counter narratives and effective solutions, we can transcend dichotomies of we vs. them, individual vs. collective, biomedical vs. humanistic, and factual vs. fictional storytelling.

## Author contributions

HW: Writing – original draft, Writing – review & editing. IC: Writing – original draft, Writing – review & editing. YS: Writing – original draft, Writing – review & editing.
